# Large language models and their performance for the diagnosis of histoplasmosis

**DOI:** 10.1371/journal.pntd.0013151

**Published:** 2025-07-09

**Authors:** Mathieu Nacher, Ugo Françoise, Antoine Adenis

**Affiliations:** 1 CIC INSERM 1424, Centre Hospitalier de Cayenne, French Guiana, France; 2 INSERM UA 17 Santé des Populations en Amazonie, Centre Hospitalier de Cayenne, French Guiana, France; Erasmus Medical Center, Rotterdam University, NETHERLANDS, KINGDOM OF THE

Progressive disseminated histoplasmosis remains a major but largely undiagnosed AIDS-defining opportunistic infection [1]. Low awareness of the disease, its proteiform presentation and the lack of rapid diagnostic tools in most endemic areas still cause potentially fatal delays in antifungal treatment [2]. This is particularly true in South and Central America where disease incidence is high but probably in Africa and Asia where it is suspected to be mostly overlooked [3–5]. In this context, Large language models (LLM) processing written case descriptions could be help avoiding missing diagnoses and reduce dangerous treatment delays [6,7]. About a year ago we published a letter on the poor capacity of CHATGPT 3.5 to identify vignettes of HIV-associated histoplasmosis—when prompted “what are the diagnostic hypotheses”, it missed 16 of 20 vignettes [8]. But since then, the capacity of AI has continued to improve and we hypothesized that the poor performance we had observed with older versions of CHATGPT may have improved. We also wished to test other LLM’s performance to identify histoplasmosis.

We thus retested different LLM’s ability to suggest a diagnosis of histoplasmosis from the same 20 clinical vignettes of people with HIV-associated histoplasmosis on which CHATGPT 3.5 had stumbled upon in early 2024. Ten of these vignettes were drawn from published case reports and 10 were from our histoplasmosis cohort (S1 Appendix) [8]. We removed the identification of *Histoplasma capsulatum* before prompting ChatGPT 3.5, ChatGPT 4.0, Microsoft copilot, Google’s AI Gemini, and Deepseek about what are the diagnostic hypotheses for the 20 clinical vignettes. We then examined the outputs to see whether histoplasmosis was mentioned as a differential diagnosis.

Thus, on March 3rd, 2025, after uploading the 20 vignettes, we prompted different LLM with “what are the diagnostic hypotheses”. The results are summarized in Fig 1 and detailed by vignette in S1 Table. ChatGPT 3.5 and 4.0 listed histoplasmosis or fungal infection among the differential diagnoses in 15 and 14 out of 20 histoplasmosis vignettes, respectively. By contrast, Gemini listed histoplasmosis for only 3 of 20 histoplasmosis vignettes. Deepseek also listed it in 3/20 histoplasmosis vignettes. We replaced all case locations by Indianapolis, a notorious histoplasmosis hotspot, to see if this impacted the results. This led CHATGPT 4.0 to revise its hypothetical diagnoses with 16 of the 20 vignettes. This was however not the case for CHATGPT 3.5, Deepseek or Gemini whose outputs looked quite stereotypical for the shorter vignettes (S1 Appendix). Surprisingly, Microsoft copilot (it is CHATGPT 4-based) listed 18 histoplasmosis and 1 invasive fungal infection for the 20 vignettes, which is still an information suggesting the need for rapid initiation of antifungal treatement. Surely, different prompts may lead to more or less alternative diagnoses and asking for the top 10 diagnoses will increase the probability of histoplasmosis to be listed [9]. However, since culture takes weeks and treatment delays may be fatal, having histoplasmosis ranked as one of a long list of diseases is not likely to translate into early treatment. By contrast, experienced clinicians from endemic areas for *Histoplasma capsulatum* will usually rapidly zoom on the diagnosis of histoplasmosis when given the same vignettes [10]. This is exactly what CHATGPT now does. CHATGPT uses Bayesian-like reasoning but does not explicitly compute probabilities. Instead, based on prior knowledge, it weighs likelihoods, and updates diagnoses dynamically when new data is introduced. Prompted whether the publication of more prevalence or incidence studies in more countries would improve CHATGPT ‘s ability to provide the correct diagnosis the answer was: “if more prevalence or incidence studies were published and incorporated into CHATGPT’s knowledge base, it would significantly enhance diagnostic accuracy by refining the prior probabilities of diseases in different regions”.

**Fig 1:**
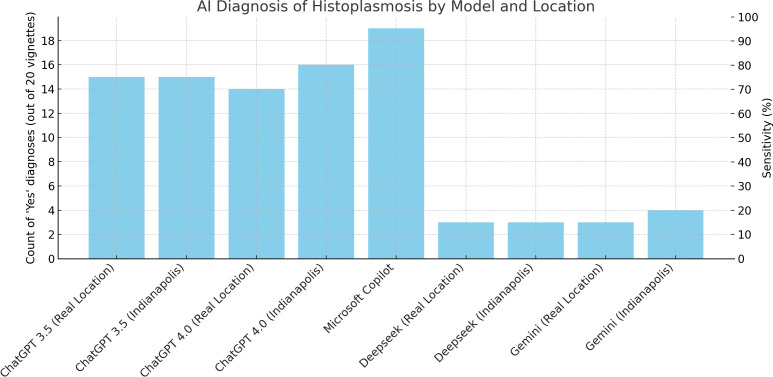
Performance of different Large Langage Models when faced with histoplasmosis vignettes and prompted with "what are the diagnostic hypotheses".

We show that, in 2025, the best results—90% sensitivity—were given by Microsoft copilot, that CHATGPT has greatly improved for identifying histoplasmosis, that it is much better than Gemini or Deepseek. Copilot features differences in training focus, fine-tuning, context handling, and safety constraints that could explain variations between different models’ diagnostic accuracy. The striking performance of Microsoft Copilot was on par with recent antigen detection tests’ sensitivity which is above 90% [11,12]. Given the rapid progress of LLM such performances should be reevaluated regularly and perhaps expanded to vignettes of persons with advanced HIV without histoplasmosis to estimate their specificity. The strength of the present data is that it uses a fixed set of histoplasmosis vignettes to compare now and then and to compare different LLM. To our knowledge, the use of LLM for the diagnosis of HIV associated histoplasmosis from clinical vignettes has not been studied. More generally, in the context of infectious disease consultations, the black box nature of LLM, their tendency to confabulation, have raised some legitimate concerns about the safety of their indiscriminate use but also recognition that this is a rapidly evolving field [13]. Others have also warned that LLM performed significantly worse than physicians and thus were not ready for autonomous clinical decision making [14]. A randomized study found that diagnostic reasoning performance was not improved by LLM [15]. Other recent studies emphasized the potential use of LLM to identify differential diagnoses and their gradual improvements [16]. However, the authors pointed that the correct responses were correlated with availability in the literature but not with actual disease incidence, which is an important perspective for improvement. Less surprisingly, others have emphasized that incorporating laboratory results improved performance [17].

How to integrate this concretely into clinical practice remains to be clarified as copilot or CHATGPT operate with written text. However, other voice-to-text software could transcribe the clinical vignette at the bedside before asking for the diagnostic hypotheses. This suggests that, as for classical grand rounds, quality clinical notes and the ability to verbalize a synthetic and accurate clinical description of cases will remain crucial skills if LLM make their way into the physician’s toolkit.

In conclusion, although we were highly skeptical a year ago about the usefulness of LLM in the very specific context of HIV-associated histoplasmosis, we must admit that a year of progress has tilted our position toward a more favorable view. We show for the first time that LLM may have potential as a point of care tool for the differential diagnosis of diseases that are neglected [18] and hard to diagnose.

## Supporting information

S1 Appendix The supplementary file lists 20 vignettes of HIV-associated disseminated histoplasmosis case reports. (DOCX)

S1 Table Is a diagnosis of histoplasmosis suggested by AI when asked to give diagnostic hypotheses? (DOCX)
