# Supplementary material for: Large language models and their performance for the diagnosis of histoplasmosis
Source: PLoS Negl Trop Dis. 2025 Jul 9;19(7):e0013151. doi: 10.1371/journal.pntd.0013151 (PMC12240382; doi:10.1371/journal.pntd.0013151)
Supplement: S1 Appendix — (DOCX) [file pntd.0013151.s001.docx]

**Vignette 1**

A previously healthy 40-year-old Japanese man presented to a community hospital in Tokyo with dry cough, fever, and progressively worsening dyspnea for 3 weeks. Chest computed tomography (CT) revealed bilateral ground-glass opacities and a 17- mm nodule in the left lower lung lobe with mild mediastinal lymphadenopathy. The cluster of differentiation (CD) 4+ T lymphocyte count was 13 cells/μL, and plasma HIV RNA copy number was 1,400,000 copies/mL. The patient worked as a consultant and had an extensive travel history. In the previous 22 years, he had visited over 30 countries in Asia, Europe, North America (including the west coast), South America, Africa, and the Middle East for business and personal purposes. His visits were mostly short-term, up to a few weeks, and in the past 3 years, mainly to Southeast Asian countries. His past medical history was unremarkable other than weight loss of 17 kg over a 2-year period. His body temperature was 36.7°C, pulse rate was 100 beats/min, blood pressure was 136/78 mmHg, respiratory rate was 16 breaths/min, and oxygen saturation was 97% in ambient air. No thrush was noted, lung sounds were clear without crackles, and no rashes were observed. The result of an interferon-gamma release assay (T-SPOT. TB) was negative. The result of a cytomegalovirus pp65 antigenemia assay was positive (293 cells per 100,000 white blood cells). Serum cryptococcal antigen was not detected. A chest radiograph showed a 20-mm nodule in the left lower lung field, with little PCP-associated pulmonary infiltration. One week after initiating antiretroviral therapy, the patient developed a fever and rashes on his face, trunk, and extremities (Fig. 1A, B). Brown macules, small papules, and erythema were observed in these areas. He had no Fig. 1. Rashes on the chest (A) and upper back (B) that developed 1 week after the initiation of antiretroviral therapy. Chest computed tomography with contrast agents 1 week after starting antiretroviral therapy shows a pulmonary nodule (arrowhead) and markedly worse mediastinal lymphadenopathy.

**Vignette 2**

A 24-year old African-American male presented complaining of 2-week history of fever, chills, and night sweats. His medical history included AIDS and herpes genitalis. His absolute CD4 lymphocyte count was 19 cells/μL with a viral load of 160,000 copies/mL. Currently he received no medications, was unemployed and lived in Georgia with no history of recent travel. Upon physical examination, the patient appeared well nourished and in no distress. The temperature was 38.2 °C, the pulse was 129, and the respiratory rate was 18/min. The blood pressure was 109/67 mmHg with an oxygen saturation of 98 percent on room air. Head, eyes, ears, nose, and throat were unremarkable except for white plaques on the soft palate. His neck was supple. Tender, warm, firm and large (2–3 cm) supraclavicular, anterior cervical, posterior cervical, submandibular, and axillary lymph nodes were noted bilaterally. The lungs were normal on auscultation. He was tachycardic with no murmurs, gallops or rubs. The abdomen was tender mostly over the right upper quadrant, and the liver edge was palpable 4 cm below the costal margin. He had enlarged and tender bilateral inguinal lymph nodes. The patient's admission laboratory results demonstrated normocytic anemia (hemoglobin 6.2 g/dL), neutropenia (absolute neutrophil count 468 cells/mm3), thrombocytopenia (platelet count 39×103/mm3), and mildly elevated transaminases (ALT 60 U/L, AST 61 U/L). Serologic tests for syphilis, hepatitis A, B, and C, toxoplasma antibody, and cryptococcal antigen were negative. The monospot test was negative, and cytomegalovirus IgM was negative. Chest Xraywas unremarkable. Three sputum samples stained for acid-fast bacilli were negative. Two sets of blood cultures were negative. Peripheral blood smear was unremarkable. Serum ferritin was greater than 20,000 ng/mL (normal 20–400 ng/mL). Both fungal and mycobacterial blood cultures were submitted to the laboratory. CT scan of the chest, abdomen, and pelvis revealed

scattered lymphadenopathy within bilateral axillar, all mediastinal stations, retroperitoneum, and pelvis. Ultimately, the patient developed respiratory distress secondary to large bilateral pleural effusions, and required non-invasive ventilatory support.

**Vignette 3**

A 30-year-old homosexual male from Trinidad and Tobago who had been in Denmark for 2

years as part of a study programme was admitted to our clinic in February 2011 with fever,

weight loss (15 kg), fatigue, night sweats, enlarged cervical lymph nodes, dyspnoea, diarrhoea and unspecific arthralgia that had persisted for several months. A colonoscopy performed before hospitalization showed diffuse inflammation of the colonic mucosa with ulceration and a suspected malignant lesion in the ileo-cecal region. Histopathological examination showed nonspecific inflammation but no sign of malignancy or granuloma formation. The patient had fever (39.0 °C) and tachycardia (120/min) at admission. Physical examination revealed cervical lymphadenopathy as the only abnormality. Laboratory results showed pancytopenia (haemoglobin 5 g/dL, white blood cells 2 × 109/L, platelets 80 × 109/L) and increased lactate dehydrogenase (LDH; 360 U/L). The patient was found to be infected with HIV-1 with severe immune suppression (CD4 T-cell count 28 cells/μL, HIV-1 plasma RNA 1,781,411 copies/mL).

A chest X-ray was normal. A positron emission tomography–computed tomography (CT)

revealed many 18-fluorodeoxyglucose (FDG)-positive lymph nodes in the neck and

retroperitoneum and diffuse increased FDG activity in the spleen. Sputum smear, culture and PCR assay for mycobacteria were negative, as were bacterial and fungal cultures from blood, respiratory samples and faeces. The interferon-release assay (QuantiFERON; Cellestis, QIAGEN, Venlo, The Netherlands) was negative. PCR analysis of induced sputum was negative for P. jirovecii. Serologic or molecular tests or both for Cryptococcus neoformans, Treponema pallidum, Chlamydia, and cultures for Neisseria gonorrhoeae were also negative. No parasites were found in the faeces.

**Vignette 4**

A 49-year-old male residing in southern Alberta presented with a two month progressive history of vomiting, diarrhea, productive cough, and shortness of breath. His history was remarkable for a 20 year history of HIV not actively on antiretroviral treatment (CD4 count of 3); housing insecurity; and lack of social supports. Travel history and infectious exposures were negative. At presentation he was tachypneic (24/min), tachycardic (112/min), hypertensive (140/94 mmHg), and febrile (Tmax 38.4 °C) but saturating well on room air (SpO2 98%). Physical examination was remarkable for cachexia including temporal wasting; bronchial breath sounds at the right lower lung base; and a diffusely tender non-peritonitic abdomen. Initial laboratory investigations including blood chemistry, complete blood count , blood gas, and coagulation panel were notable for anemia and neutropenia. Computed tomography of the thorax revealed peripheral nodularity bilaterally in the lower lung fields with some tree-in-bud patterning. Empiric treatment for Pneumocystis jiroveci pneumonia (PJP) and atypical community acquired pneumonia were initiated. Antiretrovirals were not initiated on admission. Initial bacterial blood cultures returned negative with no improvement of symptoms. Consequent bronchioalveolar lavage (BAL), urine virology, serology, and respiratory viral panel were obtained, including studies for: cryptococcus, cytomegalovirus, legionella, acid fast bacilli, and PJP. All were negative. Due to persistent febrile neutropenia he was broadened to coverage with piperacillin-tazobactam. Investigations revealed declining platelets, presence of schistocytes, elevated international normalized ratio, prolonged partial thromboplastin time, and falsely normal fibrinogen. Given his clear coagulopathy and profuse bleeding phenotype he was diagnosed with disseminated intravascular coagulation (DIC). He received tranexamic acid, as well as transfusions of packed red blood cells, fresh frozen plasma, and cryoprecipitate.

**Vignette 5**

A 37-year-old woman presented at the emergency department with following complaints: hyperthermia, cough for 15 days, weight loss and impairment of her general condition. The patient is of Cameroonian origin and arrived illegally in Belgium, which could possibly explain her late presentation to the hospital. The first laboratory results revealed severe pancytopenia (hemoglobin = 3.9 g/dL, platelets = 30,000/mm3, white blood cells = 2750/mm3) and an inflammatory syndrome with a C-reactive protein up to 180 mg/L. Among abnormal lab results, lactate dehydrogenase = 1522 U/L and ferritin = 9074 μg/L were particularly elevated. Liver enzymes were normal except for aspartate aminotransferase which was slightly elevated at 116 U/L. A positive HIV-serology and a lymphocytic typing revealed an immunosuppression compatible with AIDS (CD4 + T-lymphocyte count = 0/mm3; HIV-1 viral load = 5.3 log copies/mL). Kidney function was normal, with creatinin = 0.69 mg/dL. Ultrasound examination showed hepatosplenomegaly associated with a mild peritoneal effusion. Bone marrow aspiration pointed out a pronounced dysplasia on red cells lineage and, above all, it highlighted a significant amount of intracytoplasmic inclusions within macrophages. Subsequently, inclusions also emerged inside neutrophils on peripheral blood smears and became increasingly numerous before to progressively disappear.

**Vignette 6**

In February 2016,a 19-year-old woman coming from Ivory Coast was admitted to a peripheral hospital of our city for persistent fever. After she resulted positive for a screening HIV-Ab test, she was referred to the Infectious and Tropical Diseases Department. At admission she had deep asthenia, chills, fever (max 38.8°C), and tachycardia (110 bpm); examination revealed mono-lateral tonsillar hypertrophy and submandibular lymphadenopathy. Laboratory investigations showed pancytopenia (haemoglobin 8.7 g/dL -normal value 12.0-16.0 g/dL, white blood cells 3,050/μL -n.v. 4.00-10.80x103/μL, platelets 80,000/μL -n.v. 130-400x103/μL), increased lactate dehydrogenase (5,201 U/L -n.v. 136-234 U/L ), increased levels of C Reactive Protein (63.3 mg/L -n.v. < 5.0 mg/L ), and high ferritin and triglycerides serum levels (> 40,000 ng/mL and 440 mg/dL -n.v. 20-120 ng/mL and <150 mg/dL respectively). Baseline lymphocytes CD4+ T-cell count was 19 cell/μL and HIV-RNA was 1,787,000 cp/mL. In addition, the patient resulted positive for EBV-DNA (1,297 cp/mL), CMV-DNA (266 cp/mL), and HBV-DNA (> 700,000,000 UI/mL), and for blood stool test. Microscopical evaluation of hematoxylin and eosin (H&E) sections of the bone marrow showed a prominent lymphohistiocytic infiltrate.

**Vignette 7**

This is the case of a 29-year old male from the Peruvian jungle who was HIV positive for about 6 years, has not been receiving highly active antiretroviral (HAART) therapy, and with a CD4 count of less than 50 cells/mm3. The patient complained of diffuse abdominal pain lasting for 1 month, subsequently localized to the right lower quadrant, with an intensity of 9/10, associated with fever, chronic diarrhea, and a 6-kg weight loss. On physical examination, he looked chronically ill with pallor, and his abdomen had rebound tenderness on the right lower quadrant. Laboratory examinations showed severe hypochromic microcytic anemia, leukocytosis, hypoalbuminemia, and azotemia.

**Vignette 8**

M.Z. was a 34-year-woman, unknown HIV infected, admitted to the infectious diseases ward of Rabta Hospital of Tunis, in May 2017, two months after delivery, with a history of chronic diarrhoea and weight loss over the last 6 months. She was originating from Ivory Coast and living in Tunisia for 4 years. Physical examination found fever at 38 °C, impaired general status, global dehydration and hepatosplenomegaly. The biological evaluation showed a hypokalemia at 1.6 mmol/L, functional renal insufficiency and normochromic normocytic anemia (8.7 g/dL). HIV-Serologic test was positive, virus load was 409,500 copies/mL and CD4 T-lymphocyte count was 2 cells/mm3. Serology of hepatitis and leishmaniasis were negative. Blood cultures identified Salmonella sp. CT scan showed normal pulmonary parenchyma, necrotic intraabdominal adenopathy, peritoneal effusion, hepatomegaly and thrombosis of primary iliac venous. The diagnosis of minor salmonellosis and disseminated tuberculosis was retained. The patient received tuberculosis treatment (isoniazid-rifampicin-ethambutol-pyrazinamid) and Ceftriaxon. Seven days after, she developed a deep pancytopenia. The bone marrow smear stained with May-Grünwald stain (MGG) performed in the Laboratory of Hematology in Rabta Hospital concluded to the absence of malignancy and the presence of intracellular microorganism that do not fit with Leishmania.

**Vignette 9**

A 35 years old male farmer, from Chikamagalur, Karnataka, was referred to our tertiary care cancer hospital with a solitary oral lesion, with a suspicion of oral malignancy. He was known to be HIV seropositive, receiving anti-retroviral therapy from the local health centre for the past 3 years, and had a CD4+ T cell count of 67/μl at the time of presentation to our hospital. He presented with fever and cough of four months duration, and was on anti-tubercular therapy for the past one month after sputum revealed acid fast bacilli. The patient presented on day 0 in the out-patient services with two months history of gradually enlarging, solitary ulcer on the right lateral margin of the base of his tongue. The ulcer was 2 cm in diameter, non-tender, indurated and with rolled up margins. No significant findings could be elicited on physical examination, other than cervical lymphadenopathy. He had no history of diabetes, hypertension or asthma, or any significant travel history. He was a chronic smoker and had history of alcohol intake for twenty years. Chest radiograph done on day 2 revealed a non-homogenous opacity in right mid zone consistent with consolidation. Routine hemogram showed leucopenia with subnormal differential counts (leucocytes 2800/μl; lymphocytes 500/μl, granulocytes 1900/μl). The biochemical parameters were within normal limits.

**Vignette 10**

A 54-year-old female from Ghana, living in Italy for many years without a significant medical history, presented to the Emergency Department with syncope and persistent fever for a few days. She had not visited Africa in the past decade. On physical examination, the patient had

a temperature of 38.5 °C, pulse rate of 110/min, respiratory rate of 20/min, blood pressure of 100/60 mmHg, and 96% oxygen saturation. She had ecchymosis-like lesions on her lower limbs and postural instability, such as ataxia. Laboratory examination revealed pancytopenia: hemoglobin, 9.1 g/dl; platelet count, 112 × 103/μL; leukocyte count, 2,74 × 103/μL. The C-reactive protein (CRP) level was 80.4 (<5) mg/L, and the procalcitonin (PCT) level was 5.5 (<0.05) μg/L. She had mildly increased aspartate aminotransferase (AST) (52 U/L), alanine aminotransferase (ALT) (17 U/L), and glutamyl transpeptidase (GGT) levels (47 U/L). Furthermore, albumin was 33.0 g/L. The patient's kidney function was normal. Serum ferritin was elevated (2,997 ng/ml), and the Beta2-Microglobulin was 5.3 (range between 0.2 and 0.8) mg/L. Triglycerides were 242 mg/dl, and cholesterol levels were within the normal range. Fibrinogen and protein electrophoresis results were unremarkable, and D-dimer levels were elevated at 21,790 ng/ml (range, 0–600 ng/ml). A nasopharyngeal swab was positive for SARS-CoV-2 and negative after three days. The HIV screening test result was positive, and the patient was transferred to the Infectious Disease Department. On admission, total body computed tomography (CT) showed splenomegaly, abdominal lymphadenopathy (∅ 1.6 cm), and ventricular system of brain expansion due to cerebral atrophy. The basal HIV viral load and CD4 cell count were 1,270,000 copies/ml and 25 cells/mm3 (6%; CD4/CD8 = 0.1), respectively. Screening for opportunistic infections, including Cryptococcus neoformans serum antigen, Pneumocystis jirovecii polymerase chain reaction of induced sputum, Mycobacterium tuberculosis complex microscopy, and induced sputum polymerase chain reaction yielded negative results. Immunoglobulin G (IgG) antibodies against Toxoplasma gondii tested positive. Screening for syphilis and hepatotropic viruses yielded negative results. Low-level viremia positivity for Cytomegalovirus (CMV) and Epstein Barr virus (EBV) DNA was detected. Leishmania serology and polymerase chain reaction test results were negative. The quantiferon Tb-gold staining results were negative. Malaria was excluded. Empiric treatment for sepsis was administered without improvement in the clinical condition. Blood cultures were negative, and urine cultures revealed the growth of numerous colony-forming units of non-multidrug-resistant Escherichia coli. Lumbar puncture and brain magnetic resonance imaging MR were performed considering the objectivity of ataxia, with normal findings. Toxoplasma gondii encephalitis was excluded due to the absence of suggestive lesions. HIV genotypic resistance testing (GRT) was performed, and on day 7th antiretroviral therapy with bictegravir, tenofovir, and emtricitabine was administered after exclusion of cryptococcosis by blood serum antigen, liquor microscopy, cultural examination, and exclusion of tubercular meningitis by polymerase chain reaction and liquor microscopy. Opportunistic infection prophylaxis with trimethoprim-sulfamethoxazole (80–400 mg daily) was administered. Subsequently, GRT did not show any resistance to antiretroviral drug classes. Due to the persistence of high fever, pancytopenia, elevated ferritin levels (>8,000 ng/ml), and splenomegaly, a bone marrow biopsy was performed for the suspected hemophagocytic syndrome. Microscopic examination of the bone marrow biopsy specimen revealed myelodysplasia, probably related to HIV infection, associated with focal aspects of hemophagocytosis. Corticosteroid therapy and intravenous immunoglobulin (IVIG, 1 g/kg/day for two days) were administered without improvement. On the following days, there was a clinical condition of precipitation and a significant weight loss of >10%. The patient developed psychiatric disorders characterized by psychomotor agitation, transient hallucinations and anxiety, worsening skin lesions with scattered ecchymosis, and weight loss. Subsequent blood examinations showed further platelet reduction (20 × 103/μL), elevated PCT (17.9 μg/L), and persistently high ferritin (>8,000 ng/ml). Microbiological exams, including “T2Bacteria Panel”, blood culture and a second lumbar puncture, remained negative without microorganism growth. Another bone marrow biopsy was performed three weeks after the procedure, showing the presence of granulomas widespread hemophagocytosis.

**Vignette 11**

A 36-year-old man from Haiti, working as a gardener, with no known medical history, was admitted to the Cayenne Hospital in May 1997 for a febrile alteration of general condition, PS=3, with dry cough, dyspnea. Clinical examination also revealed centimetric cervical and axillary adenopathies. Chest X-ray revealed an interstitial syndrome. The patient also showed anemia at 9g/dl, thrombocytopenia at 108 G/L, neutrophils at 6.5 G/L, anicteric cholestasis with alkaline phosphatase 250 IU/L and GGT 200 IU/L, and CRP at 156 mg/L. HIV serology came back positive, CD4 count was 4 /μl.

**Vignette 12**

A 36-year-old man from Haiti, farm worker, with no known history of illness, was admitted to Cayenne Hospital in April 1997 for an altered general condition with febrile diarrhea, PS=2. Clinically, he presented with diffuse adenopathies, some measuring between 2 and 3 cm. Biologically, Hb was 12g/dl, platelets 322 G/L, neutrophils 1.8 G/L. He had no cytolysis or cholestasis. HIV serology was positive, CD4 count 47/μL.

**Vignette 13**

A 38-year-old Guyanese man, fisherman with a history of pulmonary tuberculosis, which did not lead to a diagnosis of HIV at the time, was admitted to the Cayenne hospital in September 2000 with febrile diarrhea, PS=2. Clinically, he presented an hepatomegaly and abdominal pain. The patient also presented with anemia at 7g/dL, platelets at 265 G/L, neutrophils at 3.9 G/l, anicteric cholestasis with alkaline phosphatase at 310 IU/L, GGT at 160 IU/l, CRP at 11 mg/L. HIV serology was positive, CD4 count 17/μl.

**Vignette 14**

A 52-year-old man, from Haiti, an agricultural labourer with no known history, was admitted to the Cayenne hospital in December 2002 with febrile dyspnoea and altered general condition, PS 3. He also presented with a dry cough and abdominal pain. Chest X-ray revealed an interstitial syndrome. He also presented with anemia at 7g/dl, thrombocytopenia at 125 G/l, neutrophils at 2.7 G/L. CRP was 98 mg/l. HIV serology was positive, CD4 count 12/μl.

**Vignette 15**

A 30-year-old woman, from Suriname, unemployed, diagnosed with HIV infection in the context of pneumocystis in 2004, but noncompliant with her antiretroviral treatment, was hospitalized in December 2007 at the Cayenne Hospital in the context of a febrile general condition, PS=4. Clinically, she presented with a productive cough, diarrhea with abdominal pain, and hepato-slenomegaly. She also presented with anemia at 10g/dL, thrombocytopenia at 59 G/L, neutrophils at 1530 G/L, hepatic cytolysis with ASAT at 139 IU/L, ALAT at 39 IU/L, CRP at 179 mg/l, ferritin at 2712 μmol/l. CD4 count was 37/μL.

**Vignette 16**

19-year-old Guyanese woman, hairdresser, with no known history, was admitted to the Cayenne hospital in August 2009 with a febrile general condition, PS=3, a weight loss of 15 kg in 6 months and fluctuating digestive disorders. Clinically, she presented with diffuse centimetric adenopathies. Alle also showed anemia at 9 g/dL, platelets at 316 G/L, neutrophils at 6.4 G/L, hepatic cytolysis with ASAT at 104 IU/l, CRP at 167 mg/l, ferritin at 894 μmol/l. HIV serology was positive, CD4 count 28/μL.

**Vignette 17**

A 37-year-old man, from Brazil, unemployed, with a history of tuberculosis leading to the diagnosis of HIV in 1999, was hospitalized in January 2011 at the Cayenne hospital for febrile dyspnea with altered general condition, PS=2, with recent weight loss of 3 kg. Clinically, she presented with diffuse centimetric adenopathy and fluctuating digestive disorders. Chest X-ray revealed an interstitial syndrome. She also presented with anemia at 10g/dL, platelets at 316 G/L, neutrophils at 9 G/L, albumin at 20 g/L, CRP at 91 mg/L and ferritin at 537 μmol/L. CD4 count was 163 μmol/L.

**Vignette 18**

A 52-year-old man, from Suriname, builder, known to be HIV-infected since 2009, was hospitalized in September 2012 at the Cayenne hospital for diarrhea with digestive pain. A chest CT scan also revealed a bilateral interstitial infiltrate. He also presented with anemia at 7g/dL, platelets at 430 G/L, neutrophils at 3 G/L, albuminemia at 16 g/L, CRP at 92 mg/L and ferritinemia at 1890 μmol/L. CD4 count was 0/μL.

**Vignette 19**

A 42-year-old man, from Suriname, gold digger, with no known history, was hospitalized in December 2015 at the Cayenne Hospital with productive cough and febrile dyspnea, abdominal pain, with altered general condition, PS=1. Clinically, he presented with diffuse centimetric adenopathies and splenomegaly. Chest CT revealed a pulmonary opacity at the right apex. He also presented with anemia at 9 g/dL, thrombocytopenia at 12 G/L, neutrophils at 0.4 G/L, hepatic cytolysis with ASAT at 128 IU/L, albumin at 29 g/L, CRP at 23 mg/L, ferritin at 29763 μmol/l and images of hemophagocytosis on the bone marrow smear. HIV serology was positive, viral load 6.7 log, CD4 count 25/μL.

**Vignette 20**

A 30-year-old man, originally from Brazil, hairdresser, with no known history, was admitted to Cayenne Hospital in July 2020 with a febrile general condition and 27 kg weight loss in 6 months. He presented with a dry cough and centimetric cervical adenopathy. The thoracic CT scan also revealed the presence of mediastinal lymph nodes. The patient also presented with anemia at 8g/dL, platelets at 310 G/L, neutrophils at 4 G/L, anicteric cholestasis with PAL at 351 IU/L and GGT at 374 IU/L, albuminemia at 19g/L, CRP at 87 mg/L, ferritinemia at 6678 μmol/l. HIV serology was positive, with a viral load of 6.3 Log and a CD4 count of 9/μl.
