# Supplementary material for: Large language models and their performance for the diagnosis of histoplasmosis
Source: PLoS Negl Trop Dis. 2025 Jul 9;19(7):e0013151. doi: 10.1371/journal.pntd.0013151 (PMC12240382; doi:10.1371/journal.pntd.0013151)
Supplement: S1 Table — (DOCX) [file pntd.0013151.s002.docx]

**Supplementary table 1. Is a diagnosis of histoplasmosis suggested by AI when asked to give diagnostic hypotheses?**

| Vignette number | CHATGPT 3.5  Real location | CHATGPT 3.5  location replaced by indianapolis | CHATGPT 4.0  Real location | CHATGPT 4.0  location replaced by indianapolis | Microsoft copilot | Deepseek  real location | Deepseek location replaced by Indianapolis | Gemini | Gemini  location replaced by Indianapolis |
| --- | --- | --- | --- | --- | --- | --- | --- | --- | --- |
| 1 | Yes | Yes | Yes | Yes | Yes | No | No | Yes | Yes |
| 2 | Yes | Yes | Yes | Yes | Yes | Yes | Yes | Yes | Fungal* |
| 3 | Yes | Yes | Yes | Yes | Yes | No | No | No | Fungal* |
| 4 | Yes | Yes | No | Yes | Yes | Yes | Yes | No | No |
| 5 | Yes | Yes | No | Yes | Yes | Yes | Yes | Yes | Yes |
| 6 | Yes | Yes | No | No | No | No | No | No | No |
| 7 | Yes | Yes | Yes | No | Yes | No | No | No | No |
| 8 | Yes | Yes | No | Yes | Yes | No | No | No | No |
| 9 | No | No | Yes | No | Yes | No | No | No | No |
| 10 | Yes | Yes | No | No | Fungal* | No | No | No | No |
| 11 | No | No | Yes | Yes | Yes | No | No | No | No |
| 12 | Yes | Yes | Fungal* | Yes | Yes | No | No | No | No |
| 13 | Yes | Yes | Yes | Yes | Yes | No | No | No | No |
| 14 | No | No | No | Yes | Yes | No | No | No | No |
| 15 | Fungal* | Fungal* | Fungal* | Yes | Yes | No | No | No | No |
| 16 | Yes | Yes | Yes | Yes | Yes | No | No | No | No |
| 17 | No | No | Yes | Yes | Yes | No | No | No | No |
| 18 | No | No | Yes | Yes | Yes | No | No | No | No |
| 19 | Yes | Yes | Yes | Yes | Yes | No | No | No | No |
| 20 | Yes | Yes | Yes | Yes | Yes | No | No | No | No |

**Fungal means the answer suggests disseminated invasive fungal infection without explicitly spelling histoplasmosis
